# GAD1 contributes to the progression and drug resistance in castration resistant prostate cancer

**DOI:** 10.1186/s12935-023-03093-4

**Published:** 2023-10-30

**Authors:** Lilin Wan, Yifan Liu, Ruiji Liu, Weipu Mao

**Affiliations:** 1 Department of Urology, People’s Hospital of Putuo District, Shanghai, 200000 China; 2 Department of Urology, Affiliated Zhongda Hospital of Southeast University, 87 Dingjia Bridge Hunan Road, Nanjing, 210009 China; 3 Southeast University, 87 Dingjia Bridge Hunan Road, Nanjing, 210009 China; 4 Department of Urology, Sichuan Provincial People’s Hospital, School of Medicine, University of Electronic Science and Technology of China, Chengdu, China

**Keywords:** GAD1, Prostate cancer, Castration resistant, Drug resistance, Immune

## Abstract

**Background:**

Prostate cancer is currently the second most lethal malignancy in men worldwide due to metastasis and invasion in advanced stages. Studies have revealed that androgen deprivation therapy can induce stable remission in patients with advanced prostate cancer, although most patients will develop castration-resistant prostate cancer (CRPC) in 1–2 years. Docetaxel and enzalutamide improve survival in patients with CRPC, although only for a short time, eventually patients develop primary or secondary resistance, causing disease progression or biochemical relapse.

**Methods:**

The gene expression profiles of docetaxel-sensitive or -resistant prostate cancer cell lines, namely GSE33455, GSE36135, GSE78201, GSE104935, and GSE143408, were sequentially analyzed for differentially expressed genes and progress-free interval significance. Subsequently, the overall survival significance and clinic-pathological features were analyzed by the R package. The implications of hub genes mutations, methylation in prostate cancer and the relationship with the tumor immune cell infiltration microenvironment were assessed with the help of cBioPortal, UALCAN and TISIDB web resources. Finally, effects of the hub genes on the progression and drug resistance in prostate cancer were explored using reverse transcription-polymerase chain reaction (RT-PCR), immunohistochemistry, cell phenotype, and drug sensitivity.

**Result:**

Glutamate decarboxylase 1 (*GAD1*) was tentatively identified by bioinformatic analysis as an hub gene for the development of drug resistance, including docetaxel and enzalutamide, in prostate cancer. Additionally, *GAD1* expression, mutation and methylation were significantly correlated with the clinicopathological features and the tumor immune microenvironment. RT-PCR, immunohistochemistry, cell phenotype and drug sensitivity experiments further demonstrated that *GAD1* promoted prostate cancer progression and decreased the therapeutic effect of docetaxel or enzalutamide.

**Conclusion:**

This research confirmed that *GAD1* was a hub gene in the progression and development of drug resistance in prostate cancer. This helped to explain prostate cancer drug resistance and provides new immune-related therapeutic targets and biomarkers for it.

**Supplementary Information:**

The online version contains supplementary material available at 10.1186/s12935-023-03093-4.

## Introduction

According to 2020 Global Cancer Statistics [1], prostate cancer has become the third most common cancer, accounting for 7.3% of new cancers, after breast cancer and lung cancer, and its mortality rate accounts for about 7% of cancer-related deaths in men worldwide [1]. The standard treatment for prostate cancer is androgen deprivation therapy, but it develops into castration-resistant prostate cancer (CRPC) after 18 to 24 months of treatment [2–4]. The two primary treatment protocols currently recommend for CRPC by the relevant guidelines are chemotherapy (docetaxel, etc.) and a novel endocrine therapy (enzalutamide, etc.) [5–7].

Docetaxel, as a taxane antimitotic drug, inhibits the androgen receptor (*AR*) transcriptional activity by promoting the binding of transcription factor forkhead box protein O1 (*FOXO1*) to *AR* promoter in the prostate cancer cell nucleus, thereby down-regulating the expression of *AR* and prostate-specific antigen (*PSA*) and ultimately promoting tumor cell apoptosis [8, 9]. Since 2004, docetaxel combined with prednisone has been the standard therapy for CRPC, which could improve the overall survival (OS) [3, 10]. Regrettably, 40–50% CRPCs respond to docetaxel, but they do not show a significant and sustained decline in *PSA*, and the median response duration is limited to 6–9 months. In addition, numerous studies have identified fatal drug resistance in patients with CRPC following docetaxel use owing to factors, including abnormal expression of tubulin-β_3_ [11], over-expression of p-glycoprotein [12], abnormal activation of hedgehog signaling pathway [13], and abnormal transcription of *AR* [14], etc.

Enzalutamide, a second-generation anti-androgen representation, more effectively preserves AR in the cytoplasm of prostate cancer cells [15]. Existing evidence indicates that most clinical CRPC occurrence is directly associated with the *AR* signaling pathway reactivation, and hence novel *AR*-targeted drugs, such as enzalutamide, have been proven to be effective in prolonging OS in patients with CRPC. Unfortunately, similar to docetaxel, the OS of patients with CRPC after enzalutamide treatment improve by only 4–6 months [16, 17]. This can be attributed to the development of enzalutamide resistance in prostate cancer due to several factors such as the emergence of *AR* splice variants [18, 19], glucocorticoid receptor expression [20], *AR* F876L domain mutation [21], and neuroendocrine differentiation [22], etc. In the face of the current situation of drug resistance caused by multiple factors, the drug’s limited therapeutic effect and the drug resistance lethality are becoming clinical challenges that need to be resolved. Nevertheless, the mechanisms of drug resistance in prostate cancer are not fully understood and there is still a lack of more precise and effective prognostic biomarkers and potential therapeutic targets.

**Fig. 1:**
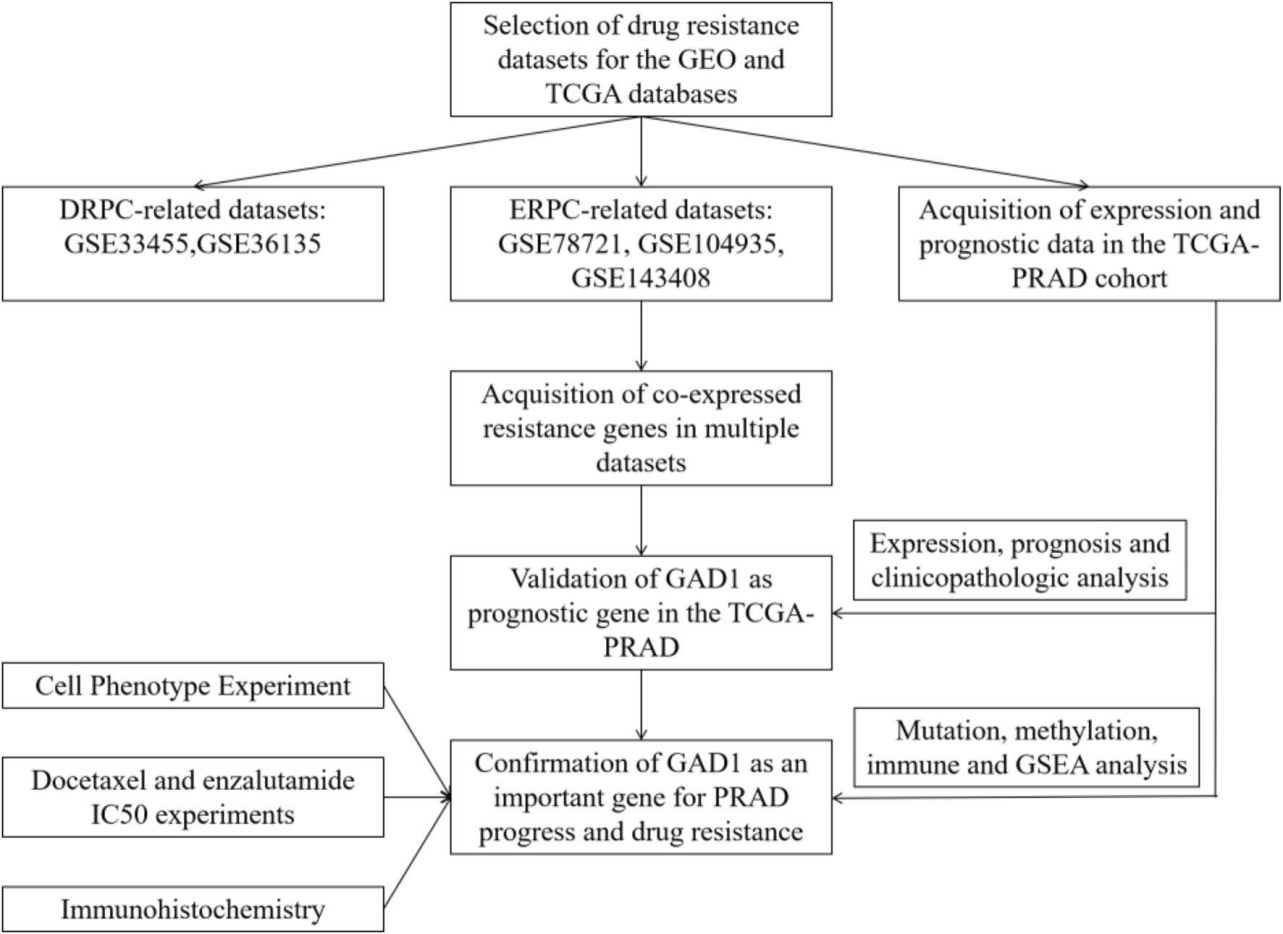
Study design flow chart

The design ideas of this study can be seen in Fig. 1. In this study, the genesets bioinformatics analysis of prostate cancer docetaxel-resistant (GSE33455 [23] and GSE36135 [24]) and enzalutamide-resistant (GSE78201 [25], GSE104935 [26] and GSE143408 [27]) are performed to identify potential target genes. The study is expected to provide insight into the molecular mechanisms of drug resistance in prostate cancer and enable the exploration of its prognostic biomarkers and potential therapeutic targets for drug resistance.

## Methods

### Microarray data analysis to screen DEGs

Based on multiple gene prostate cancer data sets, differentially expressed genes (DEGs) for drug resistance were determined by analyzing GSE33455, GSE36135, GSE78201, GSE104935 and GSE143408 datasets using R software [28] (Table 1). An adjusted p-value of < 0.05 was used as the cut-off value, and the absolute value of log-fold change |log2FC|≥ 1 was statistically significant for the DEGs.

**Table 1:** The detailed information of the two datasets

Dataset	Number of samples	Array types	Cell lines/Tissue
(Sensitive/Resistant)			
GSE33455	6 Sensitive and 6 resistant samples	GPL570 [HG-U133_Plus_2] Affymetrix Human Genome U133 Plus 2.0 Array	DU145, PC3
GSE36135	6 Sensitive and 6 resistant samples	GPL570[HG-U133_Plus_2] Affymetrix Human Genome U133 Plus 2.0 Array GPL571[HG-U133A_2] Affymetrix Human Genome U133A 2.0 Array	DU145, 22Rv1
GSE78201	12 Sensitive and 12 resistant samples	GPL10558Illumina HumanHT-12 V4.0 expression beadchip	CWP-R1, LNCaP, VCap
GSE104935	3 Sensitive and 3 resistant samples	GPL10558Illumina HumanHT-12 V4.0 expression beadchip	LNCaP
GSE143408	3 Sensitive and 3 resistant samples	GPL25684Agilent-032034 VPC Human 180 K v3 (Ensembl 77/Gencode 21/GRCh38 annotation)	LNCaP

### Hub drug resistance genes prognostic analysis

The “survival R package” was used to analyze differential expression, progress free interval (PFI), and OS of the DEGs in The Cancer Genome Atlas-Prostate Adenocarcinoma (TCGA-PRAD) cohort. Furthermore, based on the “survival R package”, the correlation between the hub drug-resistance genes and clinic-pathological characteristics of prostate cancer was investigated, and a prognostic PFI nomogram and calibration curves were developed.

### Hub resistance genes mutations and methylation analysis

cBioPortal (http://www.cbioportal.org/) clarified the glutamate decarboxylase 1 (*GAD1*) mutational profile in prostate cancer; UALCAN (http://www.ualcan.path.uab.edu/) explored the altered methylation in *GAD1*, and TISIDB (http://cis.hku.hk/TISIDB/index.php) delved into the relevance of *GAD1* expression, copy number and methylation in the tumor immune microenvironment. Subsequently, the relationship between hub drug resistance genes and immune checkpoints was analyzed by the “ggplot2 R package” and the response of hub drug-resistance genes to immune checkpoint inhibitors was assessed using algorithms for tumor mutation burden (TMB), microsatellite instability (MSI) and tumor immune dysfunction and exclusion (TIDE).

### Gene set enrichment analyses

Correlation analysis of GAD1 with all genes was conducted using Pearson’s correlation coefficients in the TCGA-PRAD. Gene set enrichment analysis (GSEA) was performed using the R package “clusterProfiler” with the following parameters: nPerm = 1000, minGSSize = 10, maxGSSize = 1000, and p-value cut off = 0.05 [29].

### Tissue samples, tissue microarrays and immunohistochemical staining

Formalin-fixed paraffin-embedded prostate cancer tissue samples were collected from patients who underwent radical prostatectomy in the affiliated Zhongda Hospital of Southeast University, China, from April 2020 to November 2021. The study samples were from patients with CRPC and the pathological diagnosis was confirmed by at least two pathologists. With the tumor as the center, normal tissues adjacent to the tumor were used as study materials and one pairs of tissue microarrays were created with a 0.6 mm diameter. For, immunohistochemistry the formalin-fixed paraffin-embedded tissue was dewaxed and dehydrated using xylene and serially-diluted ethanol. The tissue sections were incubated at 121 ℃ in an autoclave for 5 min to extract the antigen, then incubated with GAD1-monoclonal antibody (GenePharma, China) at 4 ℃ overnight, and the bound antibody (Proteintech) was incubated at 37 ℃ for 30 min. The bound antibody was detected using 3,3′-diaminobenzidine-kit and hematoxylin.

### Cell lines and cell culture

Human prostate cancer cell lines (LNCaP and PC3) were cultured with in RPMI 1640 medium (Gibco Thermo Fisher Scientific, USA), containing 10% fetal bovine serum (LONSERA, Uruguay), and 1% penicillin–streptomycin solution (Keygen, China). All cell lines were purchased from the Shanghai Institutes for Biological Sciences and incubated in 95% humidified air at 37 °C and 5% CO_2_.

### RNA extraction and RT-PCR

RNA was extracted using the RNA extraction kit (Takara Kusatsu, Japan), and Hiscript II First-Strand cDNA Synthesis Kit was used to synthesize complementary DNA (Vazyme, China). RT-PCR was performed using the MonAmp™ SYBR Green qPCR Mix (Monad Biotech, China). The primers sequences were listed in Additional file 5: Table S1. In addition, the length of the GAD1 amplicon was 170 bp.

### Small interfering RNA

The small interfering RNAs (siRNAs) of GAD1 were designed and synthesized by GenePharma Co. (China), and the sequences of siRNA was listed in the Additional file 6: Table S2.

### Cell proliferation and colony formation assays

For the cell proliferation assay, 1000 cells were seeded into 96-well plates for 0 h–120 h, and 10 µL of the cell counting kit-8 (Keygen, China) solution was added per well. After a 2 h incubation at 37 °C, optical density at 450 nm (OD 450 nm) was measured on a microplate reader (Bio-Tek, USA). For the colony formation assay, cells were seeded into 6-well plates at a density of 1–2 × 10^3^ cells/well and incubated for 10–14 d at 37 °C. Next, the cells were washed using phosphate-buffered saline, fixed with 4% polyformaldehyde (Service bio, China) and stained with 0.1% crystal violet solution (Keygen, China). Colonies containing > 50 cells were counted using the ImageJ 2X software 2.1.4.7 (Rawak Software Inc, Germany). During the experimental design, we repeated each experiment three times in order to make the results more convincing.

### Wound-healing and transwell assay

Cells were inoculated onto 6-well plates for the wound-healing assay and treated with si-/nc- GAD1. A straight scratch was made on the plate with a sterilized needle tip when the cell density was approximately 70%. The cell wound edge was marked and photographed under a microscope at the starting time point, and after 0–48 h, the cells’ migrated distance were measured and analyzed for the wound closure percentage. For transwell assays, cells were inoculated into a 24-well transwell cell apical chamber containing matrix gel (BD, USA) for evaluating invasion and gel-free for migration. The bottom and upper chambers contained the RPMI medium and fetal bovine serum-free medium, respectively. Cells that invaded the bottom chambers were fixed with 4% polyformaldehyde, stained with 0.1% crystal violet solution, counted, and photographed under a microscope. During the experimental design, we repeated each experiment three times in order to make the results more convincing.

### Statistical analysis

Statistical analysis was carried out by R software (version 4.0.2). Multivariate Cox regression analyses were used to evaluate prognostic significance. When p < 0.05 or log-rank p < 0.05, the difference was considered statistically significant.

## Result

### PARVA, ATP2B4, SH3BGRL and GAD1 as potential genes for drug resistance in prostate cancer

**Fig. 2:**
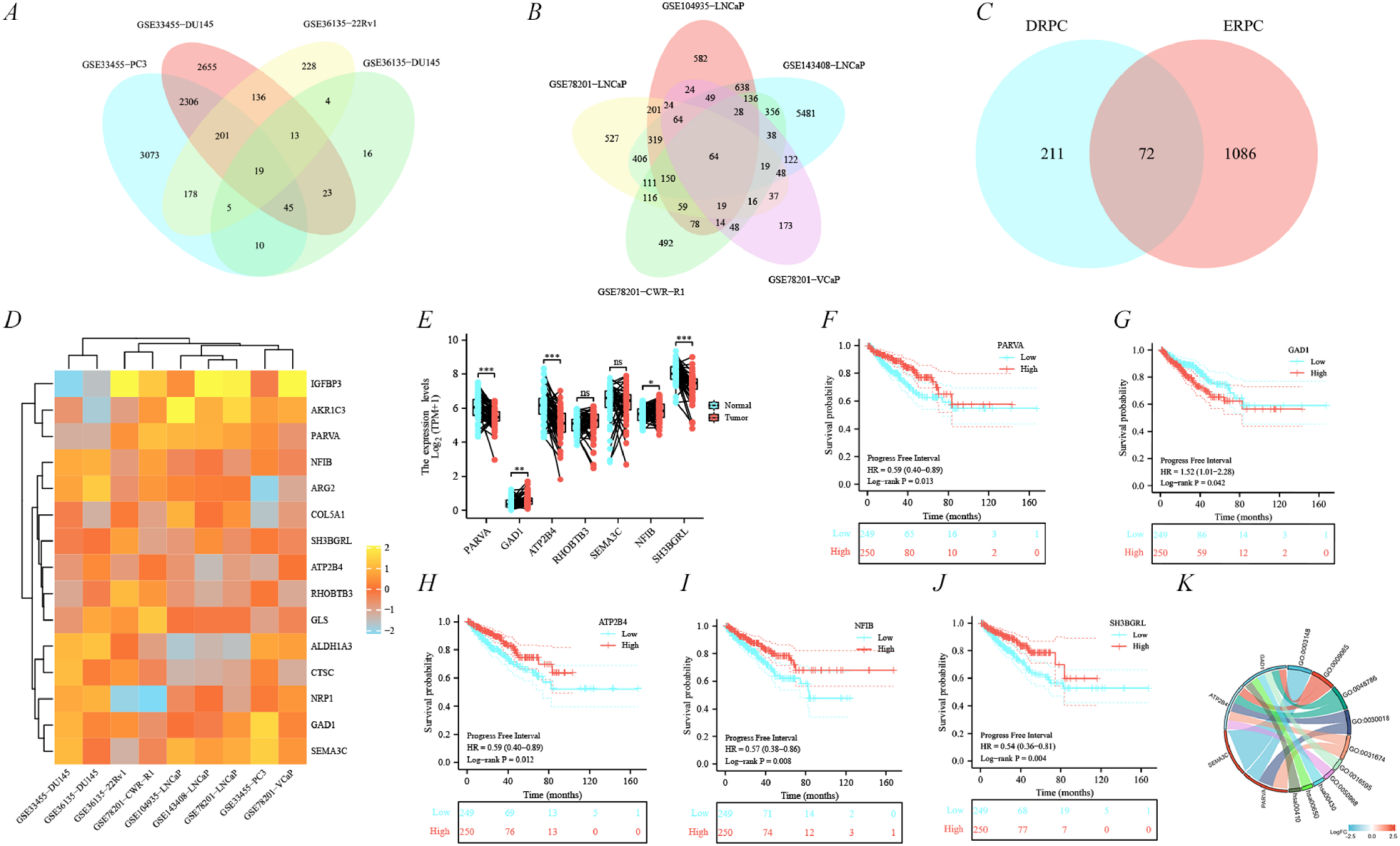
Screening for drug resistance-associated prognostic genes in prostate cancer. **A** Venn diagram of docetaxel resistance GEO dataset. **B** Venn diagram of enzalutamide resistance GEO dataset. **C** Venn diagram of drug resistance co-expression genes. **D** Heat map of drug resistance-associated prognostic genes. **E** Histogram of expression differences. **F**–**J** Progress free interval analysis, including PARVA, GAD1, ATP2B4, NFIB and SH3BGRL. **K** GO-KEGG analysis

A total of 283 prostate cancer docetaxel-resistant genes and 1158 prostate cancer enzalutamide-resistant prostate cancer genes (co-expressed in at least three cell lines subsets) were filtered from the gene expression omnibus (GEO) databases (Fig. 2A, B), and 72 co-expressed drug-resistance genes were obtained (Fig. 2C). Of these, 15 genes were initially found to be significantly associated with PFI as determined by univariate Cox regression analysis (Fig. 2D, Additional file 1: Figure S1A, B).

Subsequently after validation in the TCGA-PRAD cohort, parvin alpha (*PARVA*), ATPase plasma membrane Ca2 + transporting 4 (*ATP2B4*) and SH3 Domain Binding Glutamate Rich Protein Like (*SH3BGRL*) were identified as having low expression in prostate cancer and patients with corresponding low expression of these genes had significantly poor PFI (Fig. 2E–J). In contrast, *GAD1* has been proved to be a cancer-promoting gene (Figs. 1G, 2E). The remaining 11 genes were not considered because they were not differentially expressed in prostate cancer and para-cancerous tissues or had inconsistent expression and prognosis, including *NFIB, RHOBTB3, SEMA3C, AKRIC3, NRP1, ALDH1A3, CTSC, IGFBP3, GLS, ARG2, COL5A1* (Fig. 2E, I, Additional file 1: Fig. S1C–G). Data on valuable prognostic drug-resistant genes combined with logFC from the GEO were incorporated into the gene ontology and Kyoto encyclopedia of genes and genomes (GO-KEGG) analysis and revealed to be functionally enriched in GO:0003148 (outflow tract septum morphogenesis), GO:0009065 (glutamine family amino acid catabolic process) and GO:0048786 (presynaptic active zone) (Fig. 2K) biological processes.

### GAD1 as the hub gene affecting progression and drug resistance in prostate cancer

**Fig. 3:**
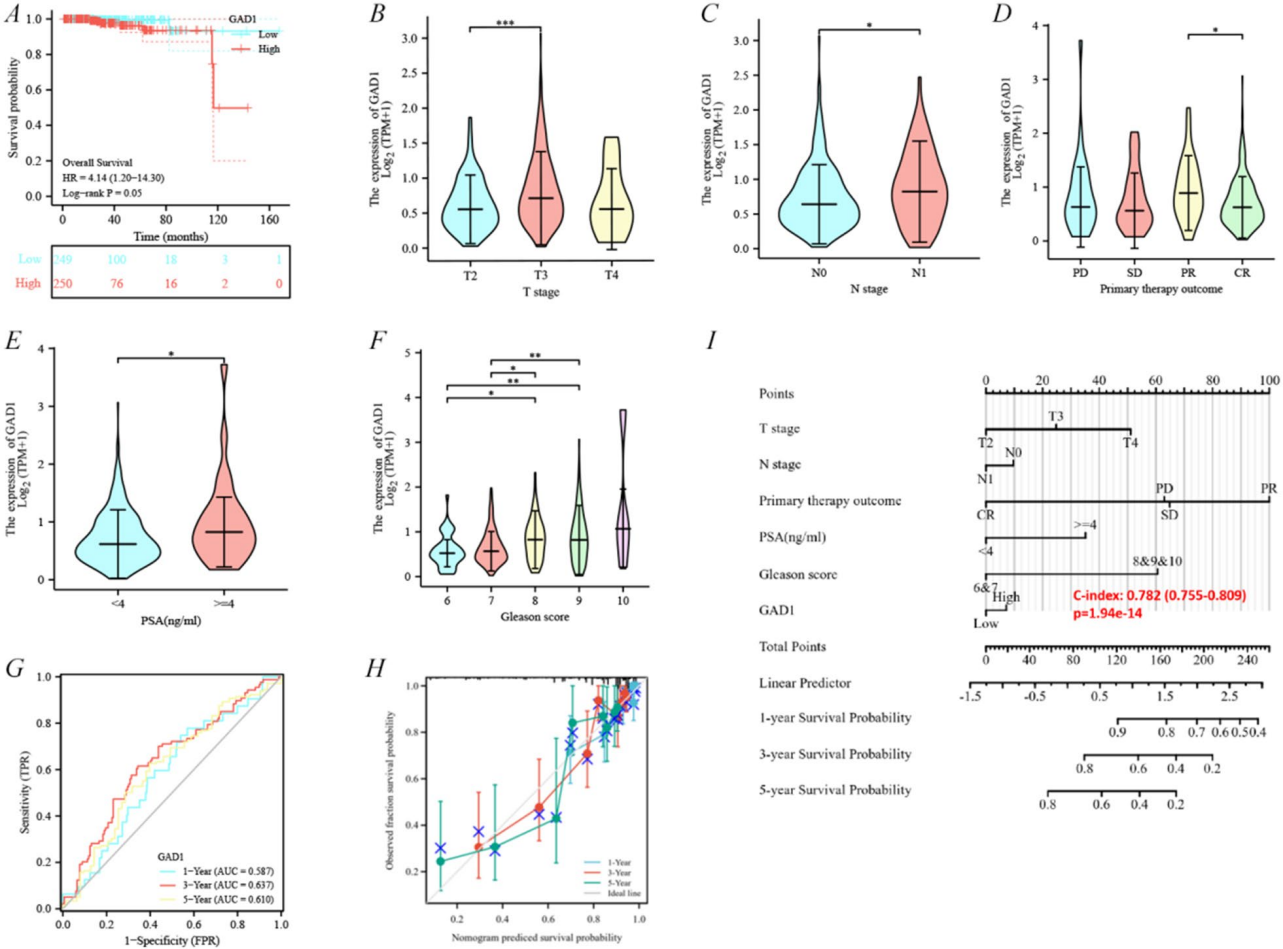
Clarifying GAD1 as a key prognostic gene in prostate cancer. **A** Overall survival analysis of GAD1. **B**–**F** GAD1 and clinicopathological features of prostate cancer, including T stage, N stage, Primary therapy outcome, PSA and Gleason score. **G** Time dependent ROC curve analyses. **H** Calibration curves of nomogram on consistency between predicted and observed 1-, 3-, and 5-year survival. **I** Nomogram for predicting 1-, 3-, and 5-year PFI in the entire TCGA cohort

To further identify the most critical drug-resistant genes, the aforementioned four genes (*PARVA, ATP2B4, SH3BGRL,* and *GAD1*) were added to the OS analysis in the TCGA-PRAD. Based on PFI, Lasso Cox regression was used to construct relevant risk prognosis models, lambda. min = 0.0015, Risk Score = (− 0.1013)*PARVA + (0.4623)*GAD1 + (− 0.1908)*ATP2B4 + (− 0.094)*SH3BGRL (Additional file 2: Fig. S2A, B). Patients were divided into a high-risk and a low-risk group according to the median risk score (50%). Survival status and hub genes heatmaps in the different groups were displayed using t-distributed stochastic neighborhood embedding and principal component analysis; results indicated that *GAD1* was highly expressed in the high-risk group, whereas *PARVA, ATP2B4* and *SH3BGRL* were expressed in low amounts in the high-risk group (Additional file 2: Fig. S2C). The prognostic model was a risk factor model owing HR = 2.216, and the median survival time of the high-risk group was significantly shorter than that of the low-risk group (p = 0.000292) (Additional file 2: Fig. S2D). Finally, we evaluated the prognostic prediction efficiency of the model by the receiver operating characteristic curve (ROC). We found that the area under the curve (AUC) was 0.611 (1-year OS), 0.660 (3-year OS) and 0.581 (5-year OS), respectively (Additional file 2: Fig. S2E). Interestingly, only *GAD1* was confirmed as the hub drug-resistant gene affecting OS in prostate cancer (Fig. 3A**, **Additional file 1: Fig. S1H–J). Moreover, the *GAD1* dryness index in prostate suggested significant differences in *GAD1* expression between the normal tissues and those with different expression levels (Additional file 3: Fig. S3), which suggested that *GAD1* may influence the degree of similarity between prostate cancer cells and stem cells, thus affecting tumor biological processes and degree of dedifferentiation.

Glutamate decarboxylase 1 (*GAD1*), a regulator of the GABA neurotransmitter metabolic pathway, is located on chromosome 2 and participates in glutamate regulation [30]. Interestingly, prostate-specific membrane antigen (*PSMA*, also called *FOLH1*), a glutamate carboxypeptidase, is also involved in glutamate metabolism and has been proven to influence the progression and metastasis of prostate cancer [31–33]. *PSMA* is a highly specific antigen and is currently widely used in conjunction with imaging for the early diagnosis of prostate cancer [34, 35]. Additionally, new targeted therapeutic agents for *PSMA* are becoming available [34, 36]. Only one study has preliminarily demonstrated using immunohistochemistry that *GAD1* is highly specific and sensitive in benign and malignant prostate tissues [37].Thus, *GAD1* is a promising candidate for the exploration of prostate cancer. In the current study, comprehensive bioinformatics analysis was performed to identify *GAD1* in many drug-resistant prostate cancer gene sets (Figs. 2, 3A).

The relationship between *GAD1* expression and prostate cancer clinic-pathological features was further investigated using the “survival R package”. Basic information about patients in the TCGA-PRAD cohort is detailed in Additional file 7: Table S3.* GAD1* expression was found to be higher in the T3 and N1 stages than in the T2 and N0 stages, respectively (Fig. 3B, C). Subsequently, the correlation of *GAD1* expression with primary therapy outcome and *PSA* was explored, and *GAD1* expression was found to be higher in *PSA* ≥ 4 ng/mL and partial response than in *PSA* < 4 ng/mL, and complete response, respectively (Fig. 3D, E). A strong correlation was found between *GAD1* expression and Gleason scores: patients with high Gleason scores had significantly higher levels of *GAD1* expression than those with low scores (Fig. 3F). Eventually, the time-dependent ROC curves of *GAD1* suggested that AUC_1-year-os_ = 0.587, AUC_3-year-os_ = 0.637 and AUC_5-year-os_ = 0.610 (Fig. 3G). Moreover, the combination of clinicopathological factors associated with *GAD1* expression in prostate cancer to create a prognostic nomogram revealed that the combined index better evaluated patients' PFI (C-index = 0.782, p = 1.94e−14) (Fig. 3I). Further, the calibration curve of the predicted probability was in good agreement with the 1-, 3- and 5-year PFI on the nomogram and the 3-year PFI was the best fit (Fig. 3H).

### GAD1 mutation and methylation associated with clinicopathological features and immune microenvironment in prostate cancer

**Fig. 4:**
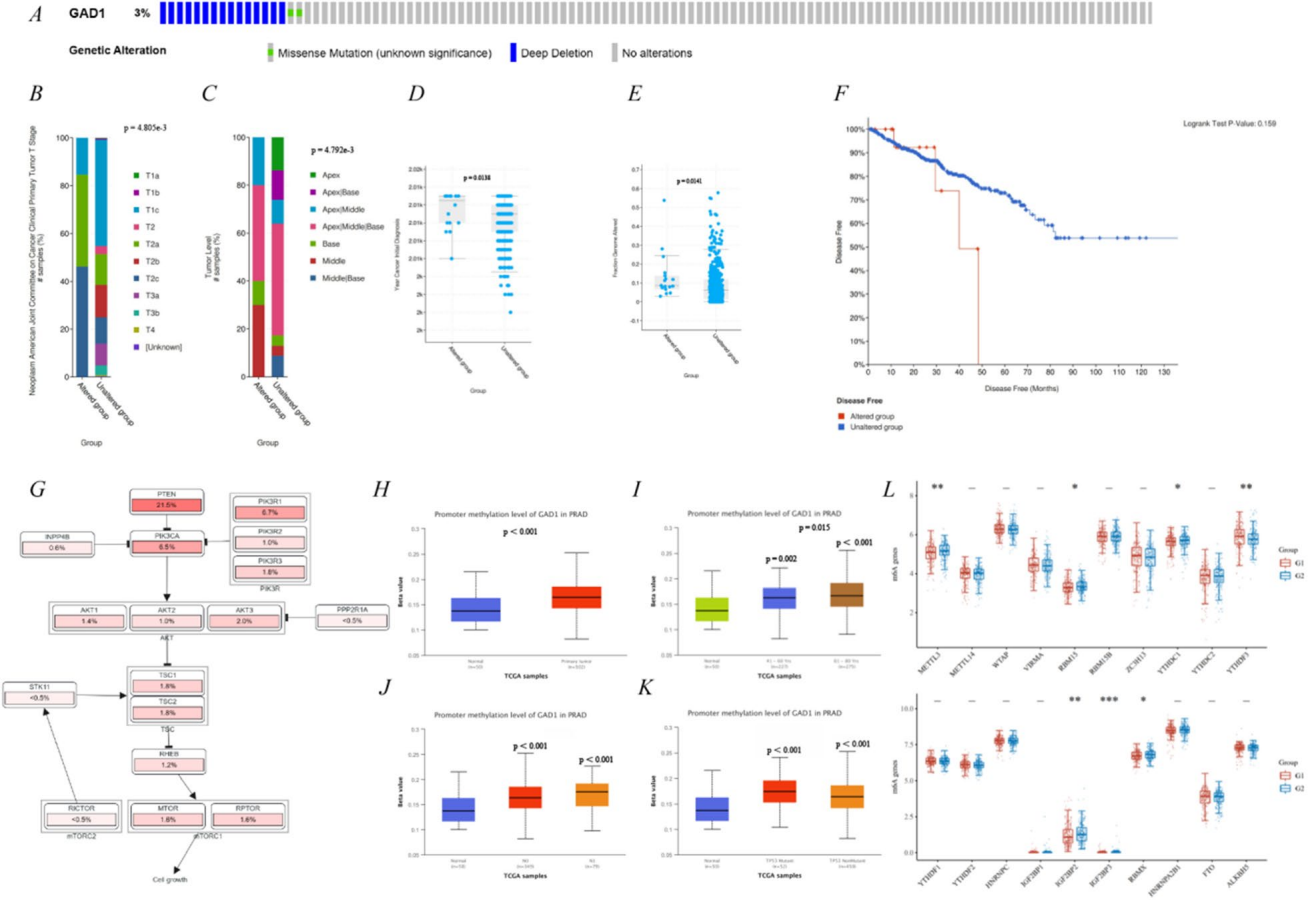
Exploring GAD1 gene mutations, methylation and clinicopathological features in prostate cancer. **A** GAD1 mutation rate. **B**–**E** Correlation between GAD1 gene mutations and clinicopathology, including T stage, Tumor level, initial diagnosis and Fraction genome altered. **F** Progression-free survival of GAD1 mutations. **G** Gene pathways influenced by GAD1 mutations. **H**–**K** Relevance of GAD1 promoter methylation to clinicopathology, including expressions, years, N stage and TP53 mutations. **L** Correlation of GAD1 with methylated genes. G1 represents GAD1-low expression and G2 represents GAD1-high expression

The above analysis initially identified *GAD1* as an important gene affecting prostate cancer progression and drug resistance. The TCGA-PRAD cohort from cBioPortal revealed that *GAD1* had a 3% mutation profile in the overall population, including deep deletion and missense mutation (Fig. 4A). Further analysis indicated that *GAD1* mutations were concentrated in the T2 stage (T2a and T2c) and middle-prostate (Fig. 4B, C), and that they were associated with a late cancer diagnosis and higher fraction genome alteration (Fig. 4D, E). However, patients with increased mutations showed a trend toward poor PFI (p = 0.159) (Fig. 4F). Additionally, the pathways most frequently associated with *GAD1* mutations was the phosphatase and tensin homolog-phosphoinositide 3-kinase pathway (Fig. 4H). Subsequently, the promoter methylation level of *GAD1* in prostate cancer was explored through the UALCAN web resource. *GAD1* promoter methylation levels were significantly higher in prostate cancer than in normal tissue and were significantly higher in older patients (Fig. 4H, I). Moreover, *GAD1* promoter methylation was significantly higher in the N0 and N1 stages than in normal tissue, but not statistically significantly different from each other (Fig. 4J). The signaling pathway for *GAD1* mutations was predominantly associated with tumor protein 53 (*TP53*); *GAD1* promoter showed higher methylation in patients with *TP53* mutated in prostate cancer (Fig. 4K). Finally, an evaluation of *GAD1* expression and methylation-related genes showed a significant correlation with methyltransferase 1 tRNA methylguanosine (*METTL1),* putative RNA-binding protein 15 *(RBM15),* YTH domain containing 1 *(YTHDC1),* YTH N6-methyladenosine RNA binding protein 3 *(YTHDF3),* insulin-like growth factor 2 mRNA-binding protein 2 *(IGF2BP2), IGF2BP3* and RNA binding motif protein X-linked (*RBMX)* genes (Fig. 4L).

**Fig. 5:**
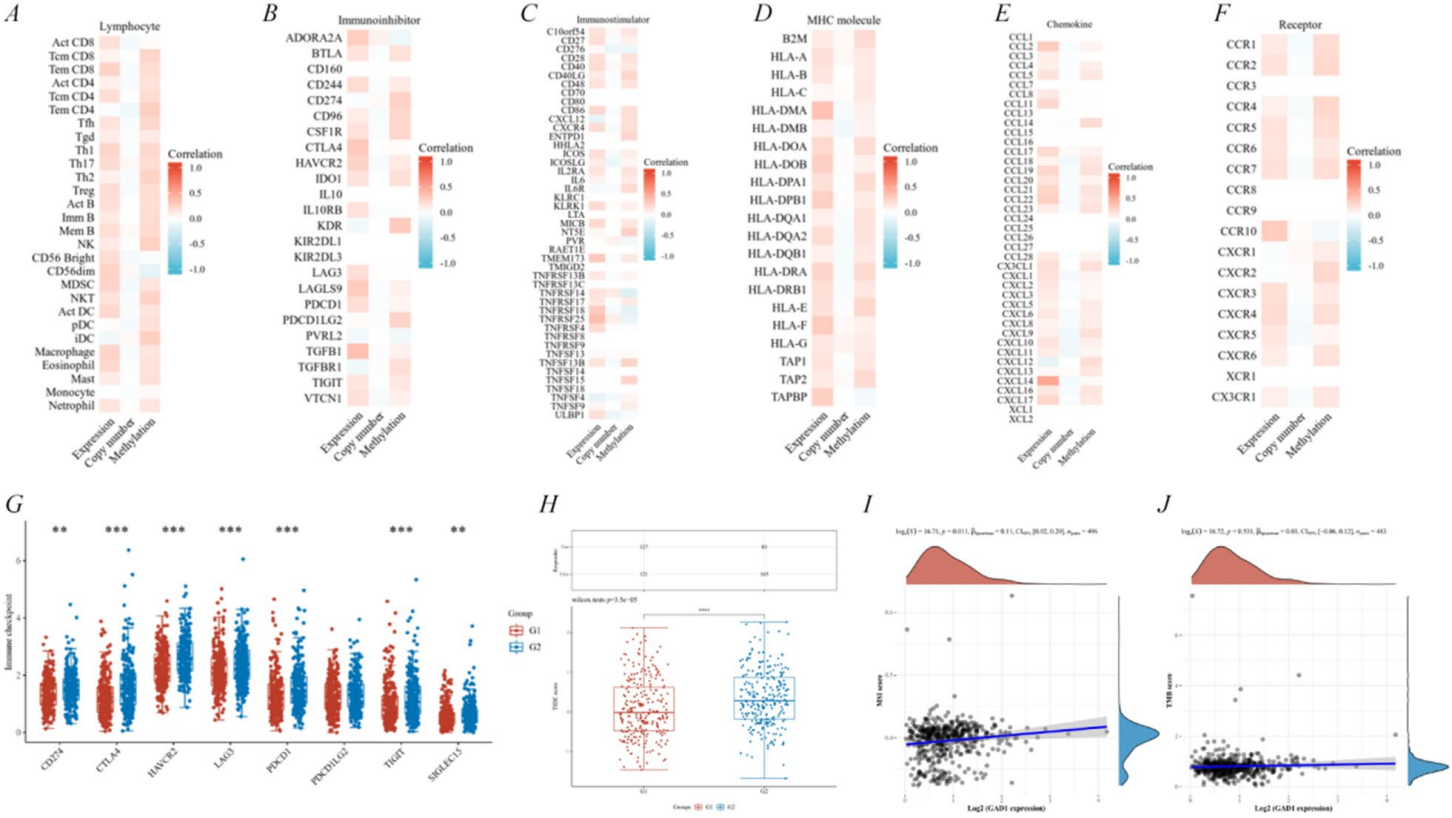
Exploiting the relevance of GAD1 to the immune microenvironment in prostate cancer. **A**–**F** Association of GAD1 expression and methylation with the immune microenvironment, including lymphocyte, immunoinhibitor, immunostimulator, MHC molecule, chemokine and chemokine receptor. **G** Correlation of GAD1 expression with immune checkpoint genes. **H** ICB score of GAD1. **I** MSI score of GAD1. **J** TMB score of GAD1. G1 represents GAD1-low expression and G2 represents GAD1-high expression

Prostate cancer, as a lymphocyte suppressive tumor, has an immune microenvironment characterized by lymphocyte deficiency and macrophage infiltration [38–41]. In recent years, immunotherapy, represented by immune checkpoint inhibitors, has achieved excellent outcomes in patients with several advanced tumors; however, prostate cancer is less responsive to immunotherapy owing to its “cold” tumor nature and low tumor mutational load [39, 40]. Consequently, whether *GAD1* can accelerate the progression of prostate cancer and the development of drug resistance by affecting the tumor immune microenvironment is an area worth exploring, and its implications for prostate cancer treatment are significant. Given the significant correlation of *GAD1* mutation and methylation with prostate cancer clinicopathology, this study also explored the interaction of *GAD1* expression, copy number and methylation with the immune microenvironment in prostate cancer. In general, with the help of the TISIDB, *GAD1* expression and methylation mostly correlated positively with lymphocyte, immunoinhibitor, immunostimulator, MHC molecule, chemokine and chemokine receptor (Fig. 5A–F). However, the *GAD1* copy number mostly did not correlate or only weakly correlated positively or negatively with the above immunological evaluation indicators (Fig. 5A–F). In prostate cancer, *GAD1* expression was significantly positively correlated with Tem CD8 (r = 0.251), Th1 (r = 0.241), CD56dim (r = 0.252), MDSC (r = 0.248) and Macrophage (r = 0.243) (Fig. 5A); *GAD1* methylation was positively correlated with Tem CD4 (r = 0.278), Th2 (r = 0.258), NK (r = 0.268) and iDC (r = 0.278), where Macrophage was not significantly correlated (r = 0.143) (Fig. 5A). *GAD1* expression was associated positively with immunoinhibitor, such as CTLA4 (r = 0.308), LAGLS9 (r = 0.296), TGFB1 (r = 0.359), and its methylation was positively correlated with CD274 (r = 0.267), KDR (r = 0.296), PDCD1LG2 (r = 0.243) (Fig. 5B). *GAD1* expression shows positive correlation with immunostimulator, such as TMEM173 (r = 0.316), TNFRSF18 (r = 0.303), TNFRSF25 (r = 0.371), and its methylation has positive correlation with NT5E (r = 0.262), TNFSF13B (r = 0.250), TNFSF15 (r = 0.243). Moreover, *GAD1* copy number was negatively correlated with CD276 (r = − 0.125), ICOSLG (r = − 0.118), and ULBP1 (r = − 0.116) (Fig. 5C). *GAD1* expression was positively related to MHC molecules, like HLA-DMA (r = 0.346), HLA-DOB (r = 0.275), HLA-F (r = 0.298), and *GAD1* methylation was positively linked to HLA-DPA1 (r = 0.203), HLA-DRA (r = 0.208), HLA-E (r = 0.229) (Fig. 5D). *GAD1* expression was positively correlated with chemokines, such as CCL2 (r = 0.289), CCL21 (r = 0.239), CXCL14 (r = 0.497), and GAD1 methylation was positively correlated with CCL14 (r = 0.207), CXCL12 (r = 0.185). Furthermore, *GAD1* copy number showed a weak negative correlation with CXCL11 (r = − 0.107) (Fig. 5E). *GAD1* expression had a positive correlation with chemokine receptors CCR10 (r = 0.291), CXCR4 (r = 0.203), and its methylation showed a positive correlation with CCR2 (r = 0.221), CCR4 (r = 0.229), CXCR2 (r = 0.218) (Fig. 5F). The above study revealed that, unlike the cold tumor characteristics, *GAD1* expression and methylation correlated significantly with both macrophages and lymphocytes in prostate cancer. Subsequently, further studies of *GAD1* in prostate cancer and paraneoplastic tissue in relation to immune checkpoint showed significant associations with CD274, CTLA4, HAVCR2, LAG3, PDCD1, TIGIT and SIGLEC15, which may lead to the speculation that they were sensitive immune checkpoints for PRAD treatment and diagnosis (Fig. 5G). Then, the Tumor Immune Dysfunction and Exclusion algorithm predicted good responsiveness to immune checkpoint inhibitors in drug-resistant prostate cancers with the low-*GAD1* genotype (p = 3.5e−05) (Fig. 5H). Further by both TMB (p = 0.531) and MSI (p = 0.011), *GAD1* was found to be associated with PD1 antibody treatment response outcomes (Fig. 5I, J). Thus, this suggests that *GAD1* may be a potential target and prognostic biomarker for immunotherapy.

### GAD1 associated with pathways of drug metabolism and immunotherapy in prostate cancers

**Fig. 6:**
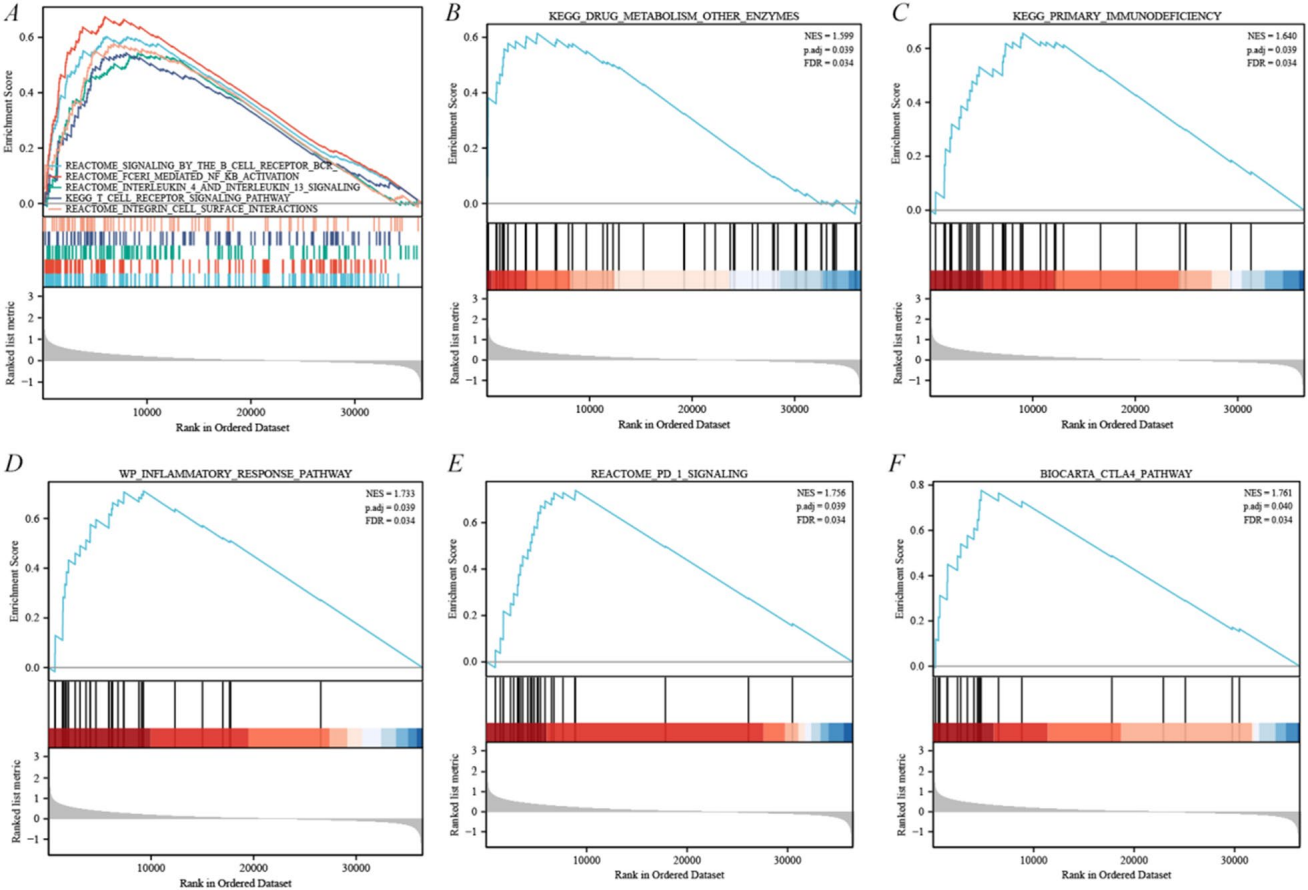
GSEA pathway enrichment analysis of GAD1

In the TCGA-PRAD cohort, *GAD1* single gene differential analysis combined with GSEA enrichment analysis revealed that pathways mainly associated with multiple immune-related pathways, such as B cell receptor, nuclear factor kappa B (NFkB) activation, interleukin (IL)-4 and IL-13, T cell receptor, integrin cell surface interactions, etc (Fig. 6A). Importantly, we found a significant correlation between the *GAD1* pathway and KEGG_DRUG_METABOLISM_OTHER_ENZYMES [normalized enrichment score (NES) = 1.599, p.adj = 0.039, false discovery rate (FDR) = 0.034], which predicted that *GAD1* might influence the metabolism of docetaxel or enzalutamide to develop drug resistance in prostate cancer (Fig. 6B). Moreover, among multiple pathways, we observed significant associations of *GAD1* with immunotherapy and immune checkpoint pathways, including KEGG_PRIMARY_IMMUNODEFICIENCY, WP_INFLAMMATORY_RESPONSE PATHWAY, REACTOME_PD_1_SIGNALING, and BIOCARTA_CTLA4_PATHWAY, all with NES > 1.600, p.adj < 0.05 and FDR < 0.05 (Fig. 6C–F). Finally, to clarify the function of *GAD1* in prostate cancer in a more comprehensive and integrated manner, we consolidated the gene ensembles of related pathways based on the work of Wei et al. [42] and calculated the enrichment scores for each sample on each pathway in turn to obtain the linkage between samples and pathways. *GAD1* was significantly involved in the gene pathway ensemble related to the following in prostate cancer: tumor inflammation (p = 3.88e−05), EMT marker (p = 1.64e−11), ECM-related gene (p = 2.92e−09), anglogenesis (p = 1.05e−08), apoptosis (p = 2.17e−08), inflammatory response (p = 1.79e−07), p53 pathway (p = 1.43e−10), TGFβ (p = 2.11E−07), IL10 anti-inflammatory signaling pathway (p = 0.001), genes up-regulated by reactive oxigen species (p = 0.001), collagen formation (p = 1.86e−16) and degradation of ECM (p = 1.14e−12) (Additional file 4: Fig. S4).

### *GAD1 affecting prostate cancer progression and drug sensitivity in *in vitro* experiments*

**Fig. 7:**
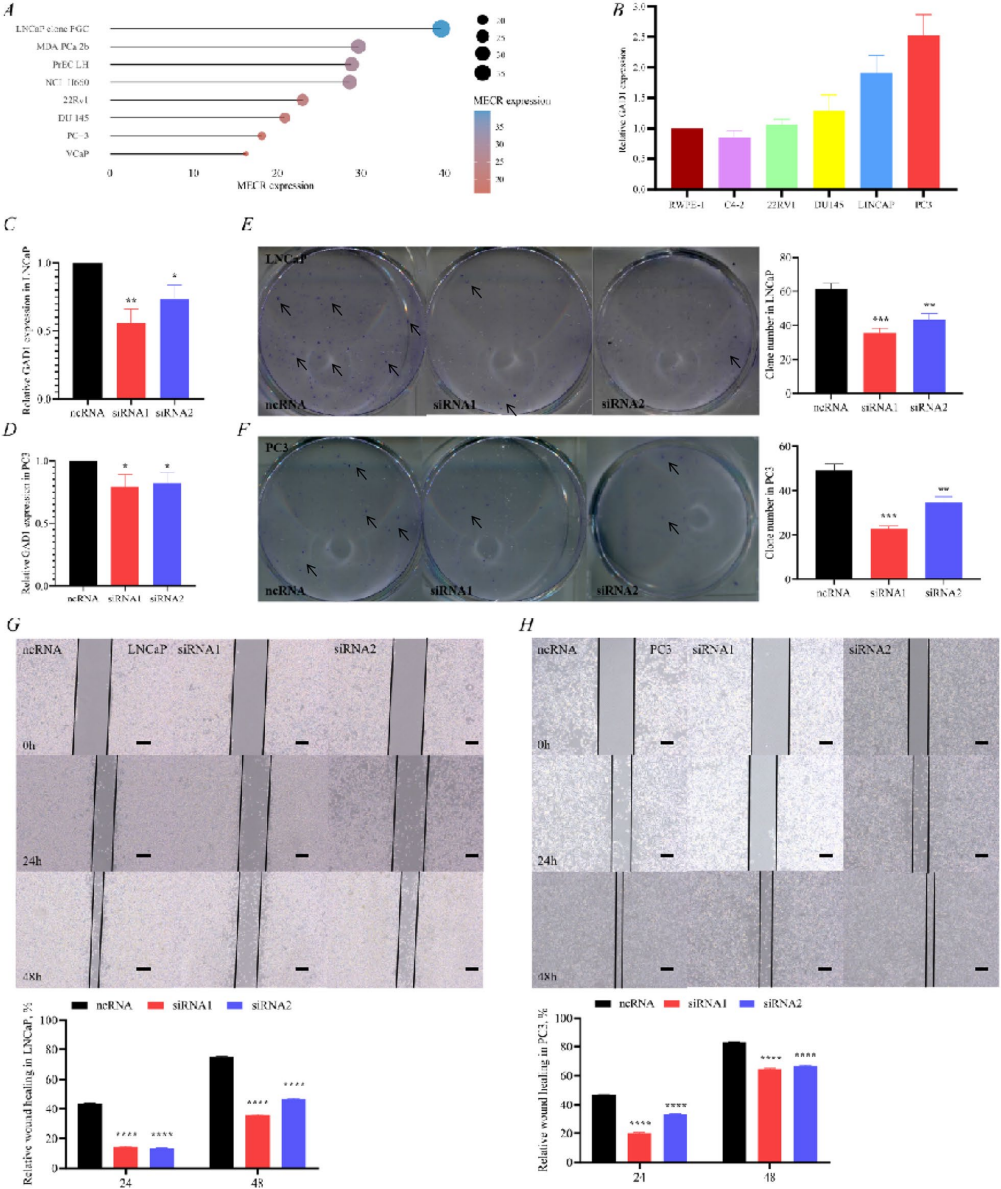

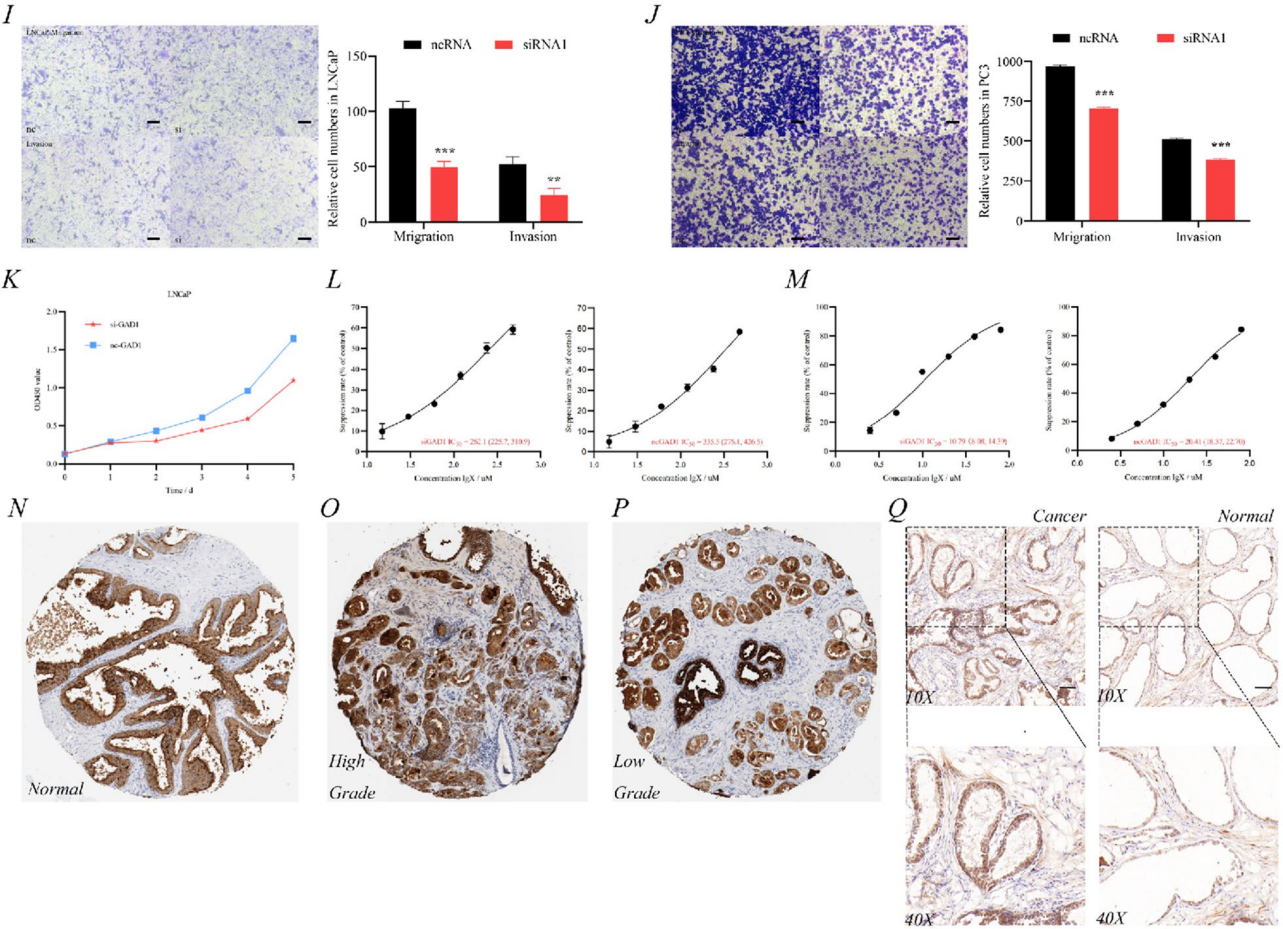
GAD1 was proved to promote prostate cancer progression and modulate drug sensitivity in vivo. **A** Expression of GAD1 in prostate cancer cell lines predicted by the CCLE database. **B** Validation of GAD1 expression in prostate cancer cell lines. **C**, **D** Knock-down efficiency of GAD1, respectively LNCaP and PC3. GAD1clone tests in LNCaP (**E**) and PC3 (**F**). GAD1 scratch tests in LNCaP (**G**) and PC3 (**H**); right, wound healing assay, scale bar, 100 μm. GAD1 was proved to promote prostate cancer progression and modulate drug sensitivity in vivo. **I** GAD1 migration and invasion assay in LNCaP in 24-well plate; right, transwell assay, scale bar, 40 μm. **J** GAD1 migration and invasion assay in PC3 in 24-well plate. **K** GAD1 CCK8 assay in LNCaP; right, transwell assay, scale bar, 40 μm. **L** Effect of GAD1 on the enzalutamide IC_50_ in LNCaP in 24 h. **M** Effect of GAD1 on the docetaxel IC_50_ in LNCaP in 24 h. HPA database demonstrates differential expression of GAD1 in normal prostate tissue (**N**), high grade (**O**) and low grade (**P**) prostate cancer. **Q** Immunohistochemistry of cancer and paracancer in prostate cancer patients at our medical centres; right, immunohistochemistry, scale bar, 100 μm

Analysis of the Cancer Cell Line Encyclopedia (CCLE) dataset (https://portals.broadinstitute.org/ccle/about) indicated high *GAD1* expression in prostate cancer cell lines NCI-H660, DU145, PrEC LH and PC3 (Fig. 7A). RT-PCR validation of the available prostate cancer cell lines in our laboratory revealed high *GAD1* expression in LNCaP and PC3 than in RWPE1 cells; thus these were selected for subsequent validation (Fig. 7B). The knock-down efficiency of the *GAD1*-small interfering reagent in LNCaP and PC3 was verified by RT-PCR (Fig. 7C, D). Subsequently, the scratch healing efficiency was lower at 24 and 48 h in the si*GAD1* group than in the nc*GAD1* group in LNCaP (Fig. 7G) and PC3 cells (Fig. 7H). In the clone assay, the number of cell clones was significantly lower in the si*GAD1* group than in the nc*GAD1* group in LNCaP (Fig. 7E) and PC3 cells (Fig. 7F). Moreover, through cell migration and invasion assays, the si*GAD1* group was shown to have a significant downregulation of cell migration and invasion ability in LNCaP (Fig. 7I) and PC3 cells (Fig. 7J). Because LNCaP cells are AR^+^ hormone-dependent prostate cancer cells, they were chosen to validate drug sensitivity. First, this study revealed that the growth curve of si*GAD1* was significantly slower than that of nc*GAD1* (Fig. 7K). Interestingly, the drug sensitivity test showed the following results: siGAD1 enzalutamide half maximal inhibitory concentration (IC50) = 262.1 μM and ncGAD1 enzalutamide IC50 = 335.5 μM (Fig. 7L). Moreover, siGAD1 docetaxel IC50 = 10.79 μM and ncGAD1 enzalutamide IC50 = 20.41 μM (Fig. 7M). These results indicated that GAD1 knockdown increased the sensitivity of prostate cancer cells to enzalutamide and docetaxel. Finally, with the help of the human protein atlas database (HPA) database (https://www.proteinatlas.org), *GAD1* expression was confirmed to show an increasing trend in prostate tissue (Fig. 7N), high-grade prostate cancer (Fig. 7O), as well as low-grade prostate cancer (Fig. 7P), which was consistent with *GAD1* expression in different grade (Fig. 3F). Immunohistochemistry of patients with prostate cancer at our medical center showed significantly higher expression of *GAD1* in prostate cancer tissue compared to that in paracancerous tissue (Fig. 7Q).

## Discussion

Prostate cancer is currently the leading malignancy that threatens men's lives, and its resistance to therapy and progression after treatment has become a pressing clinical challenge. With the advanced development of metabolomics and spatial transcriptomics, metabolic dysregulation has become a new signature in cancer. The dynamic metabolic balance between metabolic stress and tumor cell proliferation is highly dependent on the tissue environment [43], and many experiments are beginning to focus on the interaction of tumor progression, metastasis, and drug resistance with the tumor metabolic microenvironment.

The metabolic stress response of prostate cells showed unique characteristics at different stages of the disease that caused to prostate cancer progression, metastasis, and drug resistance [44]. In 2009, Prof. Chinnaiyan et al. identified sarcosine as a key metabolite in metastatic prostate cancer through metabolomic analysis, and amino acid metabolism was a marker of early tumor development [45]. Prof. Nesvizhskii et al. predicted aberrant activation of amino acid metabolism in androgen-treated LNCaP prostate cancer cells (androgen-sensitive) using paired gene expression integration analysis and proteomic data [46]. Research had revealed that androgen exposure might result in elevated amino acid metabolism and altered methylation potential in prostate cancer cells, which in turn may affect treatment sensitivity [47]. Regrettably, there are few existing studies on amino acid metabolism in prostate cancer, the mechanisms of which are still unclear and remain of great value for exploration.

In this study, bioinformatic and prognostic analyses of the GSE33455, GSE36135, GSE78201, GSE104935, and GSE143408 datasets from prostate cancer cell lines were used to identify Glutamate decarboxylase 1 (*GAD1*), which as a potential key gene affecting the prognosis, progression, and drug resistance of prostate cancer. Furthermore, by exploring gene mutations, methylation, and the tumor immune cell microenvironment, *GAD1* was shown to have an important physiological and immunological role in prostate cancer. *GAD1*, the key enzyme in the synthesis of γ-aminobutyric acid (GABA), was a regulator of the GABA neurotransmitter metabolic pathway, catalyzing the α-decarboxylation of glutamate to produce GABA [30]. As a major inhibitory neurotransmitter, GABA had many important physiological functions, such as lowering blood pressure, delaying nerve cell aging, treating psychiatric disorders, and having anti-anxiety properties [30]. Interestingly, *PSMA*, currently a hot research topic in prostate cancer, was a glutamate carboxypeptidase that acts on the C-terminus of an N-acylation-containing substrate to release glutamate [31, 33]. *PSMA* and *GAD1* were key enzymes in glutamate production and metabolism, respectively, and it is worthwhile to explore thoroughly whether *GAD1* had important functions and specificity similar to *PSMA*. Therefore, this study was the first to propose and initially demonstrate through in vitro experiments that *GAD1* influenced the progression, metastasis, and drug sensitivity of prostate cancer, which predicted an important physiological or pathological function of *GAD1* in prostate cancer.

Further, analysis of the correlation between *GAD1* and the prostate cancer immune microenvironment indicated that *GAD1* expression and methylation were significantly associated with many immune indicators, including lymphocytes, immunoinhibitors, immunostimulators, MHC molecules, chemokines and chemokine receptors. As an immune-desert tumor or “cold” tumor, prostate cancer is characterized by lymphocyte deficiency and macrophage infiltration [38–41]; moreover, various immunotherapies, including immune checkpoint inhibitors, are less effective against it [39, 40]. Further analysis revealed that *GAD1* was related to several immune checkpoints. Moreover, there was a statistically significant difference in the effect of *GAD1* expression in cancer versus its paraneoplastic expression on the immune checkpoint blockade response and MSI scores. The above results suggested that *GAD1* might inhibit immune cell function by affecting immune checkpoints, thus preventing the body from producing an effective anti-tumor immune response and causing the tumor to escape immune surveillance. Furthermore, for prostate cancer patients with high *GAD1* expression, treatment with immune checkpoint blockade and anti-programmed cell death protein 1 antibodies might be more efficacious.

Currently, *GAD1* had been shown to be associated with growth and immunosuppression in many tumors, including non-small cell lung cancer, lung adenocarcinoma, colorectal cancer, glioblastoma and others [48]. Cancer cells with aberrant *GAD1* expression had altered glutamine metabolism in non-neural tissues to synthesize the important neurotransmitter, *GABA*. *GABA* activated the *GABAB* receptor to depress *GSK-3β* activity, leading to enhanced β-catenin signaling, which ultimately caused to not only stimulation of tumor cell proliferation, but also inhibition of intratumoral infiltration of CD8 + T cells [48]. Prof. Sidonia Fagarasan discovered that B-cell derived GABA promoted the differentiation of monocytes into anti-inflammatory macrophages, secreted interleukin 10 and inhibited CD8 + T-cell cytocidal function [49]. In mice, B cell defciency or B cell-specifc inactivation of the *GABA*-generating enzyme *GAD67* (*GAD1* alleles) enhanced anti-tumour responses. Unfortunately, there were no relevant bioinformatics analyses or basic experiments to explore the potential mechanisms and functions of *GAD1* in prostate-related diseases. Prof. David Piwnica-Worms revealed that *GAD1* was expressed in CRPC cell lines, but not in androgen-responsive cell lines [50]. Using a novel fluorescence-coupled enzymatic microplate assay for *GABA* mediated through reduction of resazurin in a prostate neuroendocrine carcinoma (PNEC) cell line, acid microenvironment-induced stress increased *GABA* levels while alkaline microenvironment-induced stress decreased *GABA* through modulation of *GAD1* and glutamine synthetase (GLUL) activities. The above results demonstrated that *GAD1*-mediated *GABA* synthesis might regulate the innervation of related glands, including the prostate [50]. It is a very interesting phenomenon that *GAD1* might influence the neuroendocrinization of tumors via neurotransmitters or immunoassays, and the related mechanisms are so far unproven and deserve to be deeply explored.

This study had some limitations. First, there is heterogeneity in the results obtained from the retrospective study. Second, this study lacked in vivo data and only verified *GAD1* function in prostate cancer at the in vitro level. Third, this study did not provide insight into the specific mechanisms by which *GAD1* affects prostate cancer. Finally, more basic and large clinical trials are needed to validate the findings of this study.

## Conclusion

In this study, a key drug resistance gene of prognostic value, *GAD1*, was obtained through comprehensive bioinformatics analysis, with significant associations between mutations, methylation and the immune microenvironment. Preliminary in vitro experiments confirmed that *GAD1* promotes prostate cancer progression and metastasis, as well as down-regulates sensitivity to enzalutamide and docetaxel. Consequently, *GAD1* may serve as a new target and prognostic biomarker for prostate cancer treatment and response to drug resistance.

### Supplementary Information

**Additional file 1: Figure S1**. Expression and prognostic analysis of key resistance genes. **A**, **B** Univariate Cox regression analysis of drug resistance-associated differentially expressed genes. **C** Expression differential analysis. **D**–**G** Progress free interval, including, including AKRIC3, ALDH1A3, IGFBP3 and GLS. **H**–**J** Overall survival, including PARVA, ATP2B4 and SH3BGRL.

**Additional file 2: Figure S2**. Four drug-resistance genes accurately predict progress free interval of PRAD patients. **A**, **B** Lasso regression analysis results. **C** Risk score distribution, survival status, and expression of 4 drug-resistance genes for patients in low-and high-risk groups. **D** KM survival analyses. **E** Time dependent ROC curve analyses in TCGA set.

**Additional file 3: Figure S3**. OCLR scores of GAD1 at different expression levels in PRAD. G1 represents GAD1-low expression and G2 represents GAD1-high expression.

**Additional file 4: Figure S4**. Gene and pathway correlation analysis.

**Additional file 5: Table S1**. Sequences of primers.

**Additional file 6: Table S2**. Sequences of the small interfering RNA.

**Additional file 7: Table S3**. Basic information about TCGA-PRAD patients. Progressive disease (PD), Stable disease (SD), Partial Response (PR), Complete response (CR).

## Data Availability

Prostate epithelial cells (RWPE-1) and prostate cancer cell lines (C4-2, 22RV1, DU145, LNCaP, and PC3) were obtained from the surgical laboratory of Zhongda Hospital, Southeast University, and were provided by Professor Weipu Mao.
