# Supplementary figures and images for: GAD1 contributes to the progression and drug resistance in castration resistant prostate cancer
Source: Cancer Cell Int. 2023 Oct 30;23:255. doi: 10.1186/s12935-023-03093-4 (PMC10617133; doi:10.1186/s12935-023-03093-4)

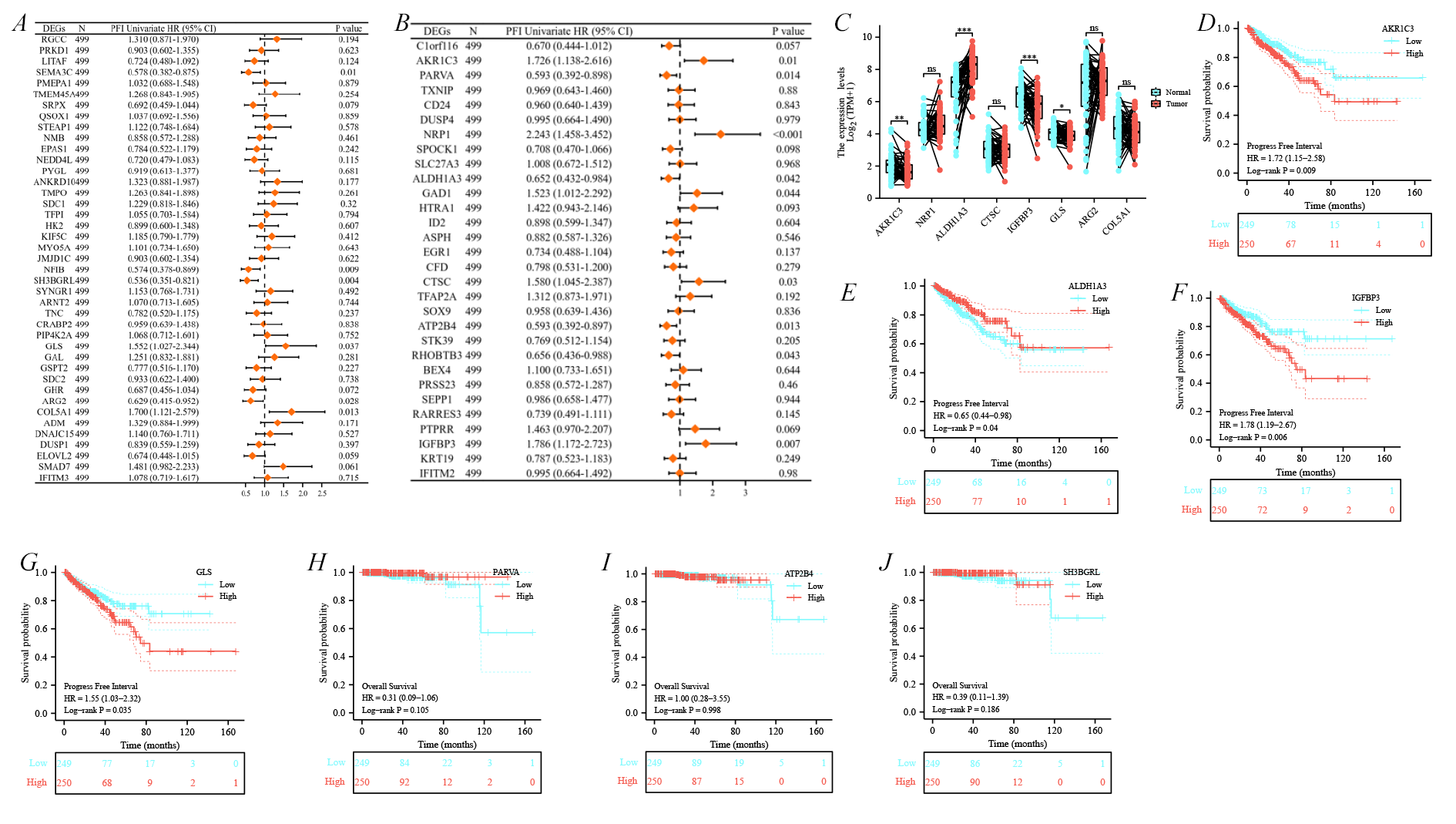

Supplement: Supplementary file 1 — Additional file 1: Figure S1. Expression and prognostic analysis of key resistance genes. A, B Univariate Cox regression analysis of drug resistance-associated differentially expressed genes. C Expression differential analysis. D–G Progress free interval, including, including AKRIC3, ALDH1A3, IGFBP3 and GLS. H–J Overall survival, including PARVA, ATP2B4 and SH3BGRL. [file 12935_2023_3093_MOESM1_ESM.tif]

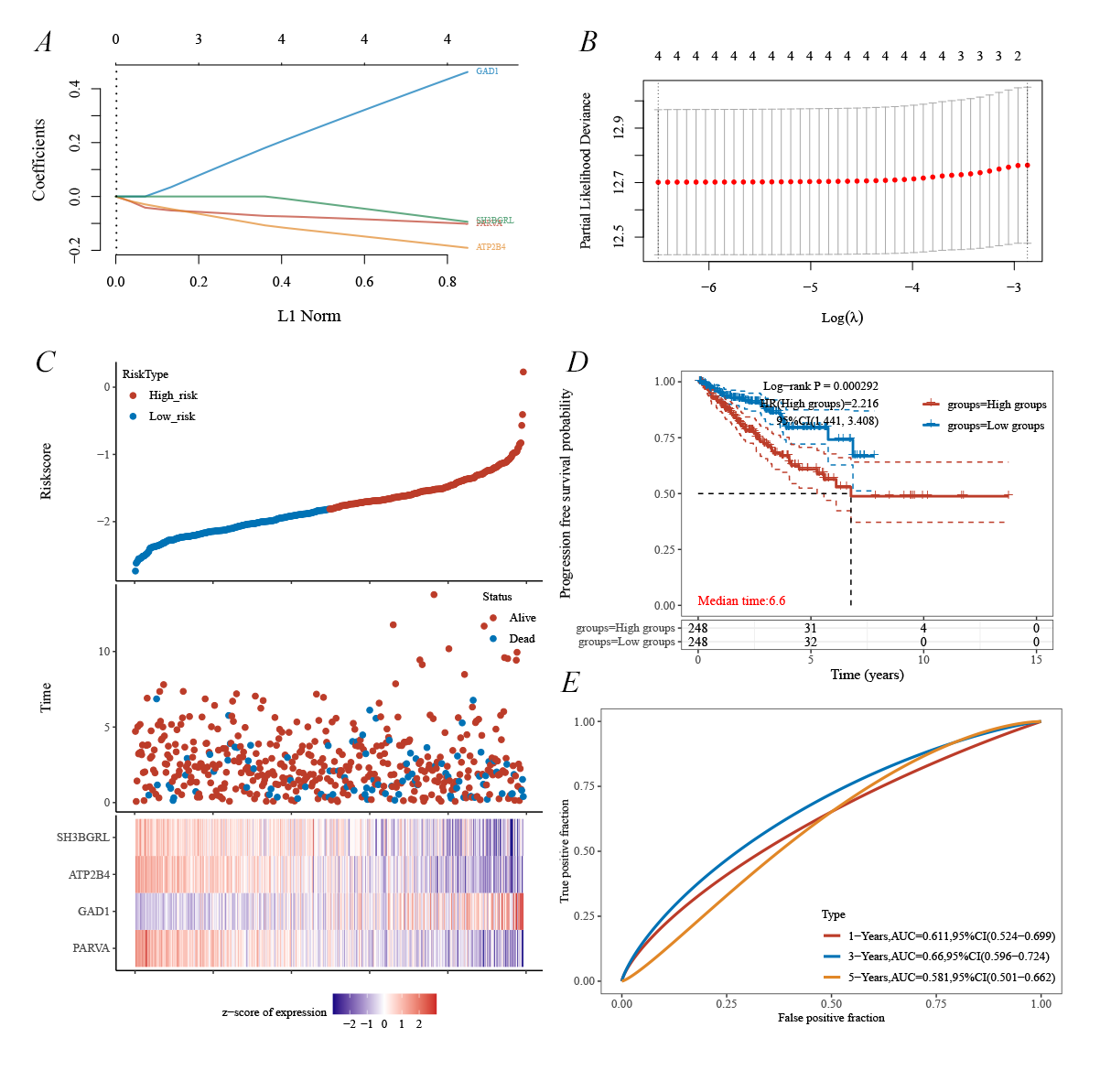

Supplement: Supplementary file 2 — Additional file 2: Figure S2. Four drug-resistance genes accurately predict progress free interval of PRAD patients. A, B Lasso regression analysis results. C Risk score distribution, survival status, and expression of 4 drug-resistance genes for patients in low-and high-risk groups. D KM survival analyses. E Time dependent ROC curve analyses in TCGA set. [file 12935_2023_3093_MOESM2_ESM.tif]

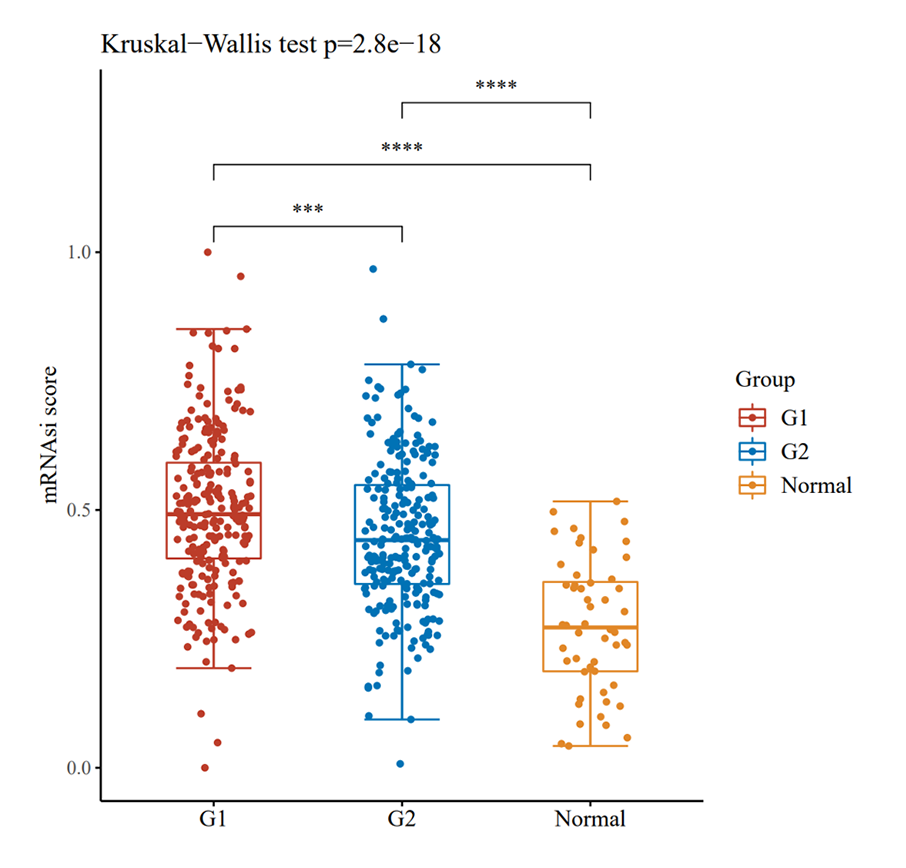

Supplement: Supplementary file 3 — Additional file 3: Figure S3. OCLR scores of GAD1 at different expression levels in PRAD. G1 represents GAD1-low expression and G2 represents GAD1-high expression. [file 12935_2023_3093_MOESM3_ESM.tif]

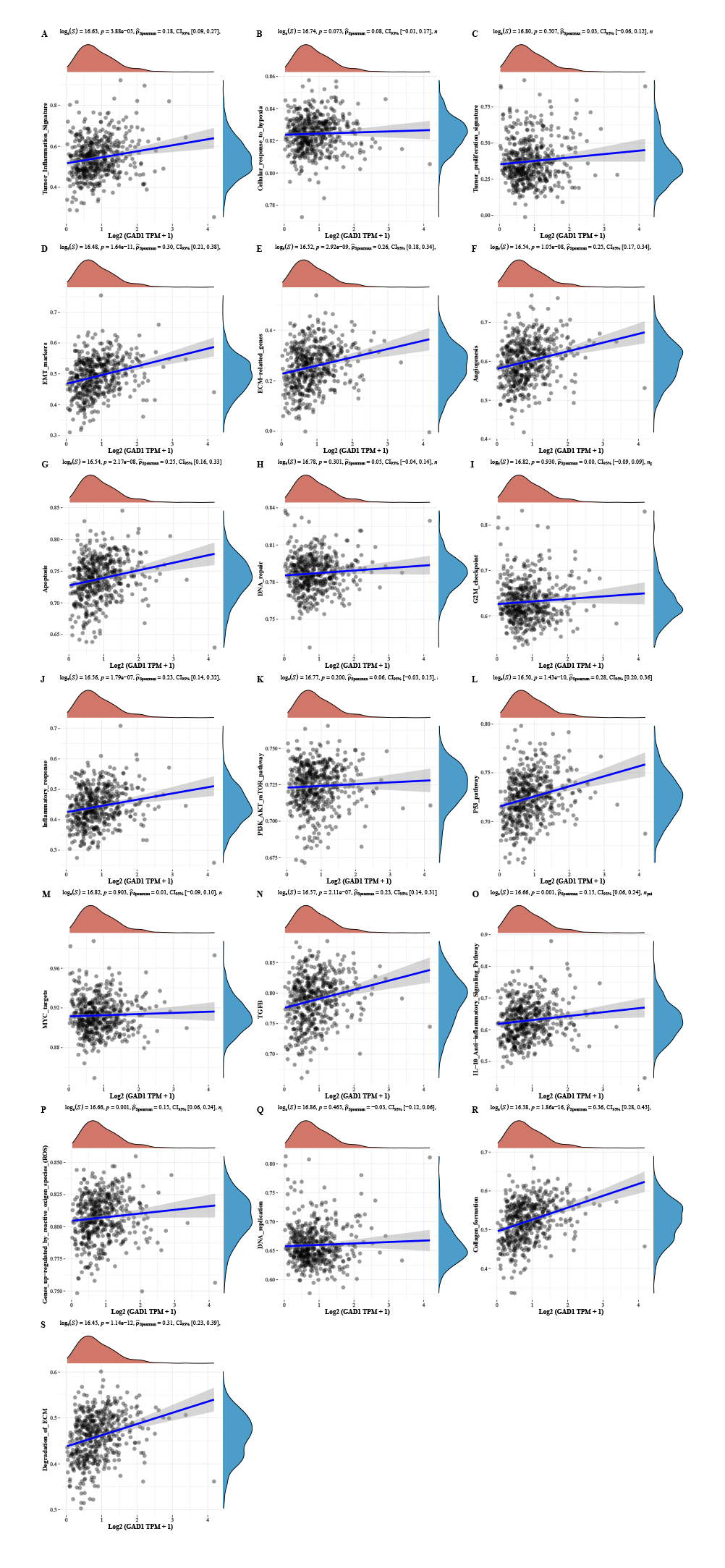

Supplement: Supplementary file 4 — Additional file 4: Figure S4. Gene and pathway correlation analysis. [file 12935_2023_3093_MOESM4_ESM.tif]
