# Supplementary material for: GAD1 contributes to the progression and drug resistance in castration resistant prostate cancer
Source: Cancer Cell Int. 2023 Oct 30;23:255. doi: 10.1186/s12935-023-03093-4 (PMC10617133; doi:10.1186/s12935-023-03093-4)
Supplement: Supplementary file 5 — Additional file 5: Table S1. Sequences of primers. [file 12935_2023_3093_MOESM5_ESM.doc]

| **Gene IDs** | **Primers** | **Sequence (5'-3')** |
| --- | --- | --- |
| 2571 | GAD1 Forward | CTGCCAACCAGCTTGTCCAAC |
|  | GAD1 Reverse | GGGAGGGTGTGACTTGTCATT |
| 2597 | GADPH Forward | GGACCTGACCTGCCGTCTAG |
|  | GADPH Reverse | GATGCCCAGGATGCCCTTGA |

**Table S1**: Sequences of primers.
