# Supplementary material for: GAD1 contributes to the progression and drug resistance in castration resistant prostate cancer
Source: Cancer Cell Int. 2023 Oct 30;23:255. doi: 10.1186/s12935-023-03093-4 (PMC10617133; doi:10.1186/s12935-023-03093-4)
Supplement: Supplementary file 6 — Additional file 6: Table S2. Sequences of the small interfering RNA. [file 12935_2023_3093_MOESM6_ESM.doc]

| The small interfering RNA | | |
| --- | --- | --- |
| siRNA1 | GAD1 Sense | GCCUUGUGAGUGCCUUCAATT |
|  | GAD1 Anti-sense | UUGAAGGCACUCACAAGGCTT |
| siRNA2 | GAD1 Sense | CAUCAACGGCCAAUACCAATT |
|  | GAD1 Anti-sense | UUGGUAUUGGCCGUUGAUGTT |
| ncRNA | GADPH Sense | UUCUCCGAACGUGUCACGUTT |
|  | GADPH Anti-sense | ACGUGACACGUUCGGAGAATT |

**Table S2**: Sequences of the small interfering RNA.
