# Supplementary material for: GAD1 contributes to the progression and drug resistance in castration resistant prostate cancer
Source: Cancer Cell Int. 2023 Oct 30;23:255. doi: 10.1186/s12935-023-03093-4 (PMC10617133; doi:10.1186/s12935-023-03093-4)
Supplement: Supplementary file 7 — Additional file 7: Table S3. Basic information about TCGA-PRAD patients. Progressive disease (PD), Stable disease (SD), Partial Response (PR), Complete response (CR). [file 12935_2023_3093_MOESM7_ESM.doc]

| Characteristic |  | | | | |
| --- | --- | --- | --- | --- | --- |
| T stage | T2 | T3 | T4 |  |  |
|  | 189 | 292 | 11 |  |  |
| N stage | N0 | N1 |  |  |  |
|  | 347 | 79 |  |  |  |
| M stage | M0 | M1 |  |  |  |
|  | 455 | 3 |  |  |  |
| Primary therapy outcome | PD | SD | PR | CR |  |
|  | 28 | 29 | 40 | 341 |  |
| Race | Asian | Black or African American | White |  |  |
|  | 12 | 57 | 415 |  |  |
| Age | <=60 | >60 |  |  |  |
|  | 224 | 275 |  |  |  |
| Residual tumor | R0 | R1 | R2 |  |  |
|  | 315 | 148 | 5 |  |  |
| Zone of origin | Central | Overlapping / Multiple | Peripheral | Transition |  |
|  | 4 | 126 | 137 | 8 |  |
| PSA (ng/ml) | <4 | >=4 |  |  |  |
|  | 415 | 27 |  |  |  |
| Gleason score | 6 | 7 | 8 | 9 | 10 |
|  | 46 | 247 | 64 | 138 | 4 |
| OS event | Alive | Dead |  |  |  |
|  | 489 | 10 |  |  |  |
| DSS event | Alive | Dead |  |  |  |
|  | 492 | 5 |  |  |  |
| PFI event | Alive | Dead |  |  |  |
|  | 405 | 94 |  |  |  |
| Age, meidan (IQR) | 62 (57, 66) | 60 (55, 66) |  |  |  |
| PSA (ng/ml), meidan (IQR) | 0.1 (0.03, 0.25) | 0.1 (0.03, 0.1) |  |  |  |

**Table S3**: Basic information about TCGA-PRAD patients.
